# *In silico* study of thymohydroquinone interaction with blood–brain barrier disrupting proteins

**DOI:** 10.2144/fsoa-2020-0115

**Published:** 2020-09-25

**Authors:** Fahad Hassan Shah, Saad Salman, Jawaria Idrees, Fariha Idrees, Muhammad Yasir Akbar

**Affiliations:** 1 Centre of Biotechnology & Microbiology, University of Peshawar, Khyber Pakhtunkhwa, Pakistan; 2 Department of Pharmacy, The University of Lahore, Islamabad Campus, Islamabad, Punjab, Pakistan; 3 Islamia College University Peshawar, Pakistan; 4 Center of Bioinformatics, Quaid e Azam University, Islamabad, Punjab, Pakistan

**Keywords:** acute toxicity, adverse effects, blood–brain barrier, CNS, molecular docking, neurodegeneration, neuropsychiatric disorders, neurotherapeutics, pharmacokinetics, thymohydroquinone

## Abstract

**Aim:**

To evaluate the inhibitory interaction of thymohydroquinone against blood–brain barrier (BBB)-associated neuropsychiatric and neurodegenerative disorders.

**Materials & methods:**

An elaborated *in silico* study was designed to evaluate the interaction of thymohydroquinone with BBB-disrupting proteins and to highlight its pharmacokinetic and safety attributes.

**Results:**

Thymohydroquinone demonstrated stable interaction with BBB-disrupting protein active site with Ki (inhibition constant) ranges of (2.71 mM–736.15 μM), binding energy (-4.3 to 5.6 Kcal/mol), ligand efficiency (-0.36 to 0.42 Kcal/mol) and root mean square deviation value of (0.80–2.59 Å).

**Conclusion:**

Further pharmacokinetic analysis revealed that thymohydroquinone is BBB and central nervous system (CNS) permeant with high acute toxicity and could be a candidate drug for the treatment of these neurological conditions.

## Lay abstract

The blood–brain barrier (BBB) is a complex neurological barrier whose disruption is associated with the development and exacerbation of different neurodegenerative and neuropsychiatric diseases. There are several drug candidates available that provide symptomatic treatment but have low BBB and central nervous system (CNS) permeability. Thymohydroquinone, a renowned medicinal compound has demonstrated a promising role in inhibiting BBB-disrupting proteins by forming hydrogen bonds with the active subunits with great stability and efficiency, thus, outcompeting its natural substrate. Through pharmacokinetic investigation, it was proven that thymohydroquinone has high BBB and CNS permeability with appropriate acute toxicity and adverse effects profiles.

In the 21st century, almost every individual is diagnosed with either neuropsychiatric or neurodegenerative disorder, thanks to the development in diagnostic medicine and genomics [1]. These disorders pose a significant threat to healthy neurological function, and may ultimately lead to numerous debilitating mental disabilities. Although etiology of these disorders remains obscure, it is assumed to be influenced by both genetic and environmental factors. These neurological anomalies are impeding economic development and further aggravating life expectancy, premature mortality and poor quality of life. Among these disorders, depression, anxiety, schizophrenia, autism, epilepsy, Alzheimer’s and Parkinson’s are largely emphasized in the research community and the development of therapeutics is underway [2]. However, the majority of drugs is utilized for symptomatic treatment but is unable to mitigate or silence the disease progression.

With the advent of increasing knowledge in genomics, numerous biological markers have been introduced, which provide effective suppression of disease pathology. However, drugs based on these markers are becoming less functional due to an insufficient understanding of disease pathology, drug resistance, inadequate drug penetrance due to the blood–brain barrier (BBB) and other elusive endogenous factors [3]. Hence, further research is required toward novel therapeutic biological markers in the brain, and drug-delivery methods to avert resistance. Despite countless methods for identifying newer biological markers and determining disease pathways, the BBB is often overlooked in neuropsychiatric and neurodegenerative disorders (NNDs). This is due to the fact that the role of the BBB in these disorders remains elusive. Although, recent studies highlighted that the disruption or leakage of these protective barriers provokes the onset of neuropsychiatric disorders, and directs the translation of pathogenic proteins, allowing toxins, immune cells and pathogens to infiltrate, leading to neurodegeneration and brain injury [4–7]. Novel biological markers that play a vital role have recently been identified, and are linked with the progression of NNDs [4,8–11]. Therefore, one could protect the neurological integrity of these barriers from these debilitating disorders by inhibiting these disease-associated markers.

The BBB is a protective element for the brain that acts as a partition between the brain’s blood vessels and glial cells and other components that constitute the brain tissue [12]. The BBB, therefore, provides a defense against disease-causing pathogens, neurotoxins and other foreign entities that are present in the blood plasma, while simultaneously allowing vital nutrients to reach the brain [6]. This barrier also maintains homeostatic hormone levels, nutrients and water in the brain to regulate its finely tuned environment. Structurally, the BBB is composed of a layer of endothelial cells which highly restrict the passage of substances from the blood [12]. As a result, the blood vessels that make up the CNS are defined as part of the BBB and are vascularized to tightly regulate the movement of ions, molecules and cells. The blood vessels and epithelial cells in the BBB communicate to change its selectivity, thus, minimizing the risk of brain infections and injury.

Instead of opting for the conventional method of targeting vital markers, the current study targets the proteins involved in deteriorating the BBB endothelium. These molecular protein markers increase BBB permeability by triggering the inflammation response, which causes immune cells and cytokines to infiltrate the barrier resulting in BBB endothelium degradation. The markers also upregulate other pathogen proteins to promote NNDs onset [4,8–11]. These molecular targets revealed a new treatment strategy for NNDs, by inhibiting these targets, one can expedite BBB rehabilitation and possibly delay or prevent the onset of these disorders.

Thymohydroquinone is a multifaceted medicinal compound that has an astounding role in alleviating various types of NNDs by inhibiting lipopolysaccharides-induced learning, memory impairments and amyloid plaque formation as well as reducing oxidative damage and detrimental hippocampal cytokine levels [13]. This compound also plays a vital role in wound healing and tissue rehabilitation; [13] however, the role of thymohydroquinone in inhibiting BBB-pathogenic proteins and BBB rehabilitation are yet to be discovered. To evaluate the inhibitory interaction of this novel drug candidate, our study aimed to observe the interaction between thymohydroquinone and BBB-disrupting protein. An elaborated drug repurposing study was designed to determine the molecular inhibitory mechanism of thymohydroquinone against BBB-disrupting proteins.

## Materials & methods

### Retrieval & preparation of ligand & receptors

In this study, the thymohydroquinone ligand was retrieved from PubChem (CID 95779) and corresponding receptors for which the activity of this ligand was investigated were human IFN-γ (1HIG), resisitin (1RFX), IL-1β (1T4Q), TNF-α (1TNF), VCAM-1 (1VSC) and MMP-9 (6ESM), respectively. The ligand and receptors were subjected to energy minimization to fulfill the prerequisites for molecular docking analysis by utilizing the PRODRG server [14] and Modrefiner [15].

### Active site determination of target receptors

The active site of these receptors was determined using Metapocket [16] to identify potential ligand-binding residues and to focus the ligand toward these active target sites. The energy-minimized receptors were uploaded to the database and three active sites were obtained (Supplementary Table 2) from which the most optimal active site was selected to observe the molecular interaction between the ligand and these active sites.

### Molecular docking & stability analysis

The receptor–ligand interaction was performed with reliable and accurate molecular docking software known as Autodock 4. The receptor and ligands were prepared as pdbqt files (Supplementary Table 3) and grid-box for site-specific docking was specified to the ligand for interaction (Supplementary Table 1). The docking results were obtained by selecting the top-scoring dock poses with strong binding affinity, which were subsequently visualized to observe ligand–receptor hydrogen interaction and to obtain their structural and molecular images. The ligand was further checked for stability studies using root mean square deviation (RMSD) through the LigRMSD database [17].

### Pharmacokinetics, acute toxicity & prediction of adverse effects

**Figure 1.**
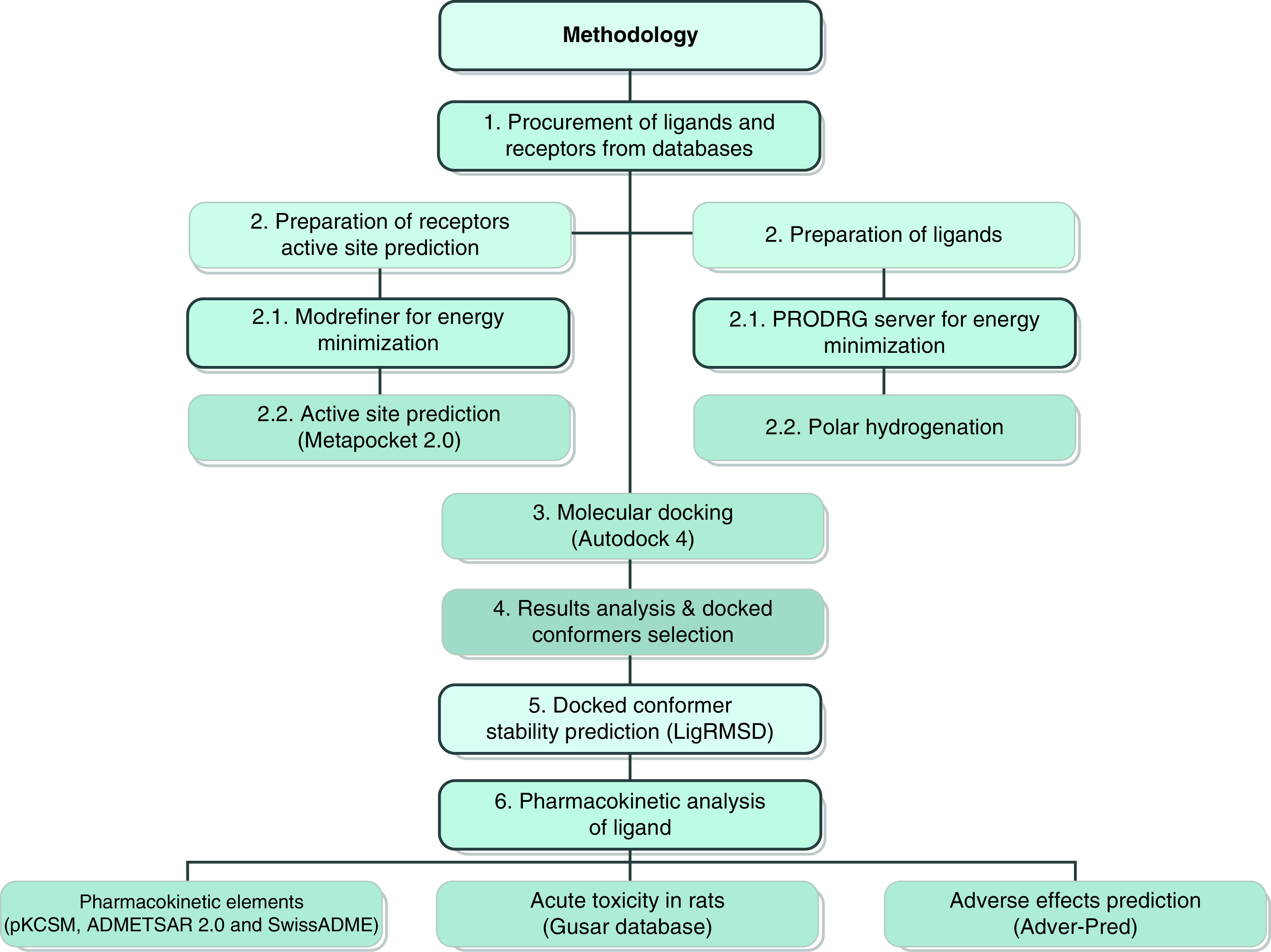
Schematic illustration of methodology of current research design.

The acute toxicity of thymohydroquinone was elucidated through the GUSAR database [18] and its pharmacokinetic attributes were predicted with the help of PkCSM [19], SwissADME [20] and ADMETSAR 2.0 [21] to confirm whether this selected ligand is permeable to BBB or not. ADVERPred [22] was used to identify possible adverse effects that may occur during administration. Such predictions were made by procuring their canonical script from PubChem and pasting it on the appropriate dialogue box to carry out their scrutiny in these stated databases. The entire methodology is summarized in Figure 1.

## Results

### Docking & stability analysis

Docking studies were performed with Autodock 4, which allows for accurate and effective protein–ligand interaction modeling. The RMSD analysis was facilitated by LigRMSD [17] to cross-validate docked structural conformations with the experimentally solved structures. The interaction between thymohydroquinone and its target receptors were evaluated. These structures include human IFN-γ (1HIG), which disrupts tight vascular junctions [23], resisitin (1RFX) which contributes oxidative stress and inflammation and increases BBB endothelial permeability) [24], IL-1β (1T4Q) which augments BBB permeability and impairs astrocytes functions [25], TNF-α (1TNF) which disrupts tight junctions [26], VCAM-1 (1VSC) which provides attachment sites for cytokines and chemokines to induce inflammatory lesions [27] and MMP-9 (6ESM) which triggers neuroinflammation and degradation of BBB-regulating proteins [28]. These proteins are involved in the BBB structural integrity, resulting in neurodegeneration and onset of various neuropsychiatric disorders [8,9,29].

**Figure 2.**
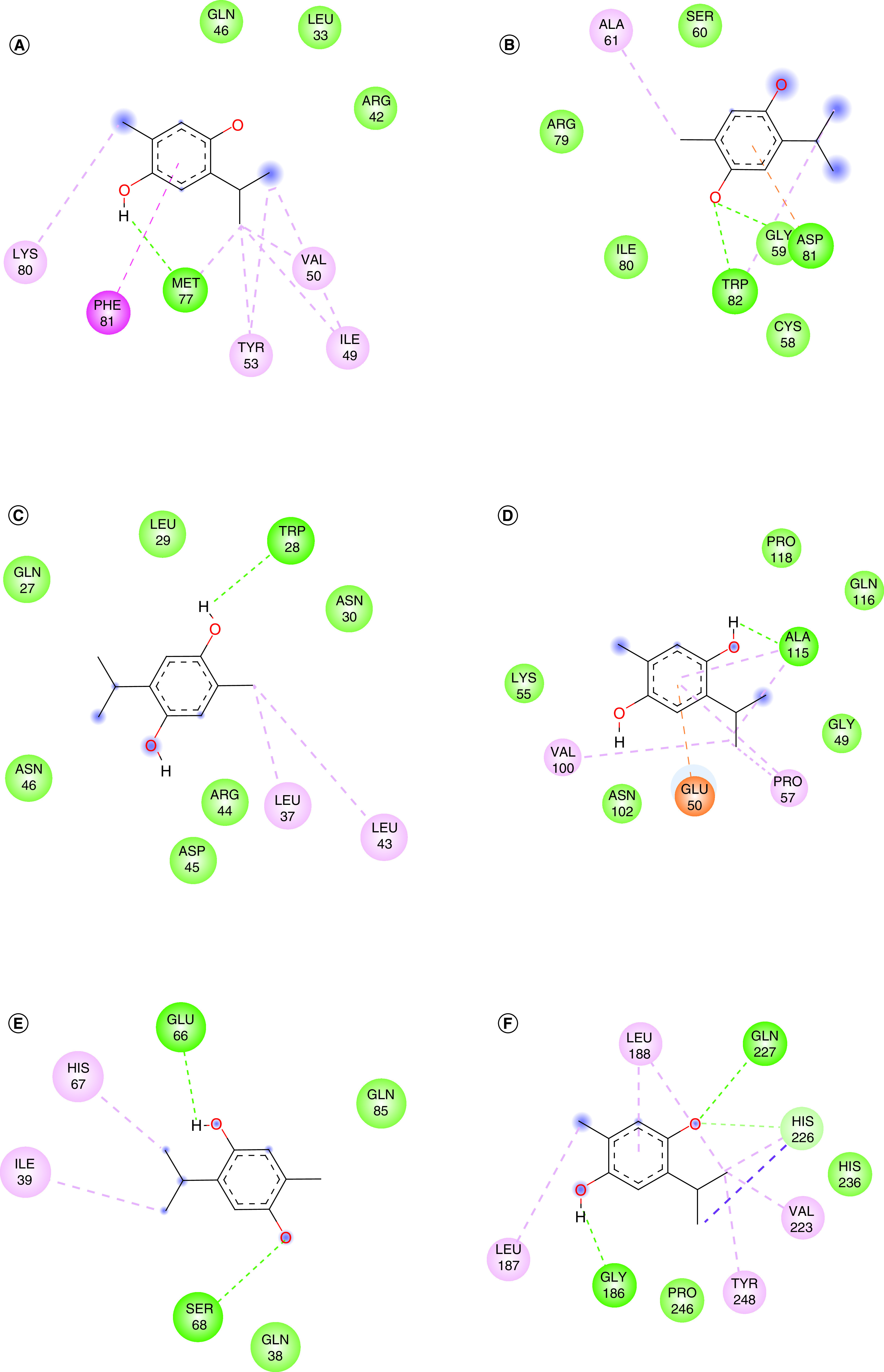
Amino acid participating in interaction with thymohydroquinone. **(A)** Human IFN-γ. **(B)** Resisitin. **(C)** IL-1β F101W. **(D)** TNF-α. **(E)** VCAM-1. **(F)** MMP-9. Green line depicts hydrogen bond, pink line depicts amide-Pi stacking, purple line depicts Pi-sigma bond and light yellow line depicts Pi-sulfur interaction.

**Figure 3.**
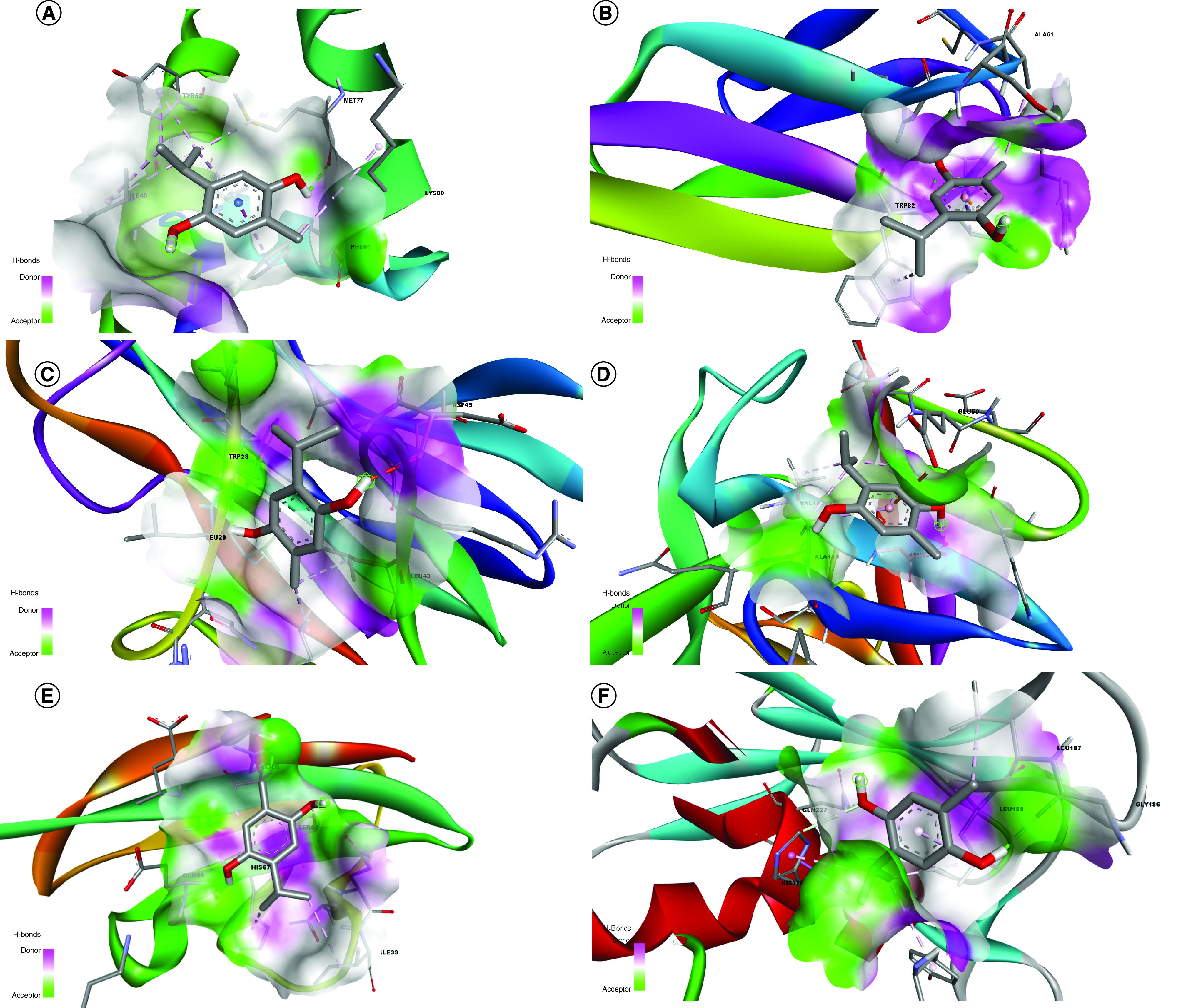
Molecular representation of thymohydroquinone interaction with active site of target proteins. **(A)** Human IFN-γ. **(B)** Resisitin. **(C)** IL-1β F101W. **(D)** TNF-α. **(E)** VCAM-1. **(F)** MMP-9.

The target receptors and ligand were prepared and refined for docking interaction using Modrefiner [15] and PRODRG [14]. These refined structures were subsequently employed to predict potential ligand-binding sites, facilitated by Metapocket 2.0 [16]. Docking parameters were established by programming the ligand toward the active ligand-binding site to determine probable interaction with the target receptors (Supplementary Table 1). The docking studies were characterized based on the nature and strength of molecular interaction as described by binding energy, ligand efficiency and inhibition constant. These characteristics reflect the strength and stability of various molecular interactions of the ligand with the target receptor. The results of protein–ligand interaction were resolved based on participation in hydrogen bond formation with the active site of target receptors. It was observed that thymohydroquinone established a single hydrogen bond with the active site of the inflammatory proteins such as human IFN-γ, IL-1β and TNF-α, whereas double hydrogen bonds are formed with resisitin, VCAM-1 and MMP-9 (Figure 2). The active amino acid residues participated in hydrogen bond formation with the ligand were MET77, ASP81, TRP28 and TRP82, ALA115, GLU66, SER68, GLY186, and GLN227, respectively. The binding energy and RMSD recorded for ligand–receptor interaction were -4.3 to 5.1 kcal/mol and 1–2 Å as summarized in Table 1 and Figure 3. The ligand efficiency ranged within -0.3 and 0.46 Kcal/mol and the maximum inhibition constant for the resisitin–thymohydroquinone complex, are given in Table 1.

**Table 1.** Elaborated profile of thymohydroquinone interaction with selected protein targets.

n	Receptor	Ligand	Predicted active site subunits	Active subunits in H-bond formation	Inhibition constant value	Binding energy (Kcal/mol)	Ligand efficiency (Kcal/mol)	LigRMSD analysis (Å)
1	Human IFN-γ (1HIG)	THQ	SER47, PHE81, PHE92, LYS88, PHE82, ASP91, LEU28, PHE29, PHE52, LEU56, PHE15, PHE60, TYR53, LEU11, PHE57, LYS43, ILE44, ASN25, SER20, ASN16, SER40, GLN46, ASP21, GLY26, THR27, LEU30, LYS12, ILE73, HIS19, GLY18, GLY31, ILE49, LEU33, VAL50, **MET77**, ASN83, ALA17, LYS13, ALA8, GLU39, LYS80, VAL22, ARG42, ASN85, ASP76, ASN78, GLU9, VAL79, TYR14, TYR4, VAL5, GLN1, GLN48, SER51, THR72	**MET77**	736.15 μM	-5.1	-0.42	0.80
2	Resisitin (1RFX)		GLY59, **ASP81, TRP82**, SER60, CYS58, ALA61, ARG79, ILE80, CYS62	**ASP81, TRP82**	2.71 mM	-4.3	-0.29	2.59
3	IL-1β F101W (1T4Q)		GLN48, LYS94, MET95, GLY49, LYS97, VAL100, **ALA115**, GLU50, PRO57, ASN102, GLU96, GLN116, PRO118, SER114, GLU113, ARG98, PHE117, GLU51, LYS55, SER52, VAL47, ASN119, TRP101, ILE104	**ALA115**	102.68 μM	-5.6	-0.41	1.82
4	TNF-α (1TNF)		VAL16, **TRP28**, LEU29, ASN30, LEU37, LEU43, ASN46, GLN47, ARG31, GLN27, LEU26, ARG44, ASP45, LEU48, LEU132, SER133, ALA134, PHE152, SER81, LYS90, GLU135	**TRP28**	435.46 μM	-4.7	-0.36	1.25
5	VCAM-1 (1VSC)		LEU12, GLU87, ILE88, PRO120, ARG123, ILE177, ARG10, ASP178, GLU179, MET180, ASP181, THR185, TYR89, PHE91, LEU175, HIS176, VAL183, PRO184, TYR11, **GLU66**, HIS67, **SER68**, GLN85, VAL86, TYR119, ARG146, LYS147, ASP122, PHE121, ASP143, ASP145, SER148, LEU149, PRO7, ALA13, GLN14, SER182	**GLU66, SER68**	394.50 μM	-4.3	-0.38	1.49
6	MMP-9 (6ESM)		HIS190, ALA191, PRO193, GLN227, HIS230, HIS236, HIS226, TYR179, ALA189, PRO246, LEU187, PRO240, GLU241, ALA242, LEU243, MET244, TYR245, MET247, ARG249, TYR248, LEU222, VAL223, LEU188, GLY186, PHE250, TYR218, ASP185, PHE221, THR251, GLU252, GLY253, ARG143, LEU212, LYS214, SER219, PRO254, PRO255, ASP139, ALA140, VAL136, LEU234, ASP235	**GLY186, GLN227**	417.94 μM	-5.5	-0.41	1.13

H bond: Hydrogen bond; RMSD: Root mean square deviation.

The bold letters signifies active amino acids that showed Hydrogen interaction with the ligand.

### Toxicity & adverse effects

Toxicity studies were performed in order to determine the lethal dose of thymohydroquinone evaluated through different administration routes in rodent models. Possible adverse effects were also predicted. Thymohydroquinone elicits a lethal response at a dose of 431,600 mg/kg through intraperitoneal administration; 81,660 mg/kg for intravenous; 838,600 mg/kg for oral; 471,700 mg/kg subcutaneously as given in Table 2. It is classified as a class 4 chemical according to OECD classification. ADVERPred results revealed that thymohydroquinone induces hepatotoxicity as evident from their value Pa >0.7; with 0.7 as the threshold for safe dosage. Other rare side effects are arrhythmia and cardiac failure.

**Table 2.** Predicted lethal dosage and adverse effects of thymohydroquinone.

Compound	Administration route	Log_10_ (mmol/kg)	(mg/kg)	OECD chemical classification	Adverse effects
Thymohydroquinone	Intraperitoneal	0.414	431,600	Class 4	Hepatotoxicity, arrythmia, cardiac failure
	Intravenous	-0.309	81,660	Class 4	
	Oral	0.703	838,600	Class 4	
	Subcutaneous	0.453	471,700	Class 4	

OECD: Organisation For Economic Co-operation and Development.

### Compound pharmacokinetics

The nature and behavior of compound under consideration were evaluated to confirm its effectiveness in averting failures in drug development and clinical and animal studies. Thymohydroquinone is readily absorbed by the gastrointestinal tract, has high water solubility and skin permeability. This compound has high CNS and BBB permeability, resulting in the ability to bypass drug-hindering barriers and exert therapeutic activity against various NNDs. CYP450 1A2 is inhibited by thymohydroquinone that prevents drug interactions and increases its drug half-life. It has a total clearance value of 0.24 and minimal toxic and carcinogenic properties. The pharmacokinetics of thymohydroquinone is given in Table 3.

**Table 3.** Pharmacokinetic analysis of thymohydroquinone.

ADMET parameters	Compounds
	Thymohydroquinone
**Absorption**	
Human intestinal absorption	90.669% absorbed
Human oral bioavailability	High
Caco-2 permeability (log Papp in 10–6 cm/s)	1.549
Water solubility (log mol/l)	-1.839 (soluble)
Subcellular localization	Mitochondria
Skin permeability (Log Kp)	-2.729 cm/s
**Distribution**	
P-glycoprotein substrate	Yes
P-glycoprotein I inhibitor	No
P-glycoprotein II inhibitor	No
BBB permeability (log BB)	0.111 (yes)
CNS permeability (log PS)	-1.738 (yes)
**Metabolism**	
CYP2D6 substrate	No
CYP3A4 substrate	No
CYP1A2 inhibitor	Yes
CYP2C19 inhibitor	Yes
CYP2C9 inhibitor	Yes
CYP2D6 inhibitor	No
CYP3A4 inhibitor	No
**Excretion**	
Total clearance (log ml/min/kg)	0.24
Renal OCT2 substrate	Yes
**Toxicity**	
Ames toxicity	No
Hepatotoxicity	No
*hERG* inhibition	No
Eye irritation	No
Carcinogenicity	No
**Druglikness and bioavailability score**	
Lipinski	Yes; 0 violation
Bioavailability score	0.55

BBB: Blood–brain barrier.

## Discussion

The prevalence of NNDs is drastically rising and heavily imposing global economic burdens. There are symptomatic treatments available for these disorders, but many have already developed resistance [30]. Moreover, the medications approved by the US FDA and other regulatory authorities incite adverse effects upon consumption [31,32]. These include DNA polymerase damage [33], developmental retardation, pseudotumor cerebri, glaucoma and other fatal syndromes [31,32].

In the past few years, the pathology to drug discovery approach revealed promising new molecular drug targets that are highly efficacious and prevent disorder onset. These molecular targets include human IFN-γ, IL-1β, TNF-α which incite an exaggerated inflammatory response by degrading the intricate basement membrane structure [23,25,26], resisitin destroys microvascular endothelial cells by oxidative stress and inflammation [24], VCAM-1 provides attachment sites to various immune cells and cytokines surrounding the barrier to degrade their tight junction [27] andMMP-9 instigates demyelination, amyloid plaques formation and digests microvascular structures leading to leaky and disrupted barriers [28]. These protein factors are recognized for the development of these disorders by damaging BBB endothelium and associated proteins and repairing cellular organelles causing irreversible colossal damage. Also, BBB leakage allows entry of various pathogens, toxins and other foreign entities along with immunoinflitration which further contributes toward neurodegeneration and onset of the neuropsychiatric disorders [8–11]. The deterioration of BBB is the primary indication of the development of these brain affecting disorders [4].

Several drug candidates are focused on targeting proteins that are present after or during BBB deterioration which may be involved in drug failure toward alleviating disease symptoms. On the other hand, several drug candidates have been proposed through virtual screening and molecular docking methods [34–36]. In these studies, the compounds show favorable interaction with the pathogenic proteins but possess low BBB and CNS permeability, high toxicity and adverse effects. Therefore, further interventions are required to counteract the adverse effects and deliver these drugs to visceral regions of the CNS.

Medicinal plants contain several compounds that were exploited to rejuvenate mental and cognitive decline; most are BBB and CNS permeant and have low toxicity as evidenced by recent literature [37]. Thymohydroquinone is derived from the *Nigella sativa* plant, and is renowned for its myriad of biological activities, including neuroprotective and antidepressant effects [13]. However, the role of thymohydroquinone toward BBB-disrupting proteins is still obscure and was predicted in this article via molecular docking using Autodock 4.

Structural irregularities, unwanted contacts and unstable potential energy were eradicated from receptor–ligand models using Modrefiner [15] and PRODRG [14] to evaluate their interaction as closely to the cellular environment as possible. Molecular docking analysis showed that oxygen and hydroxyl functional groups in thymohydroquinone participated in hydrogen bonding with the active site interface of these target receptors. Thus, these hydrogen bonds augment drug efficiency and therapeutic inhibitory action [38]. Other docking parameters were also considered to evaluate binding energy, ligand efficiency and inhibition constant. These parameters reflect the thermodynamics of interaction and indicate the potency necessary to inhibit these proteins. The binding energy and ligand efficiency range from -3.5 to 5.1 Kcal/mol and -0.29 to 0.42 Kcal/mol, respectively. The inhibition constant values range from 100 to 736.15 μM except for the resisitin–thymohydroquinone complex which is recorded to be 2.71 mM. Furthermore, the RMSD value fluctuates between 0.80 and 2.59 Å which indicates higher docked complex stability [39]. Pharmacokinetic elements and adverse effects were also predicted to inform clinicians and ensure safety during administration in different animal and clinical trials. The acute toxicity of thymohydroquinone in rodent models was >400,000 mg/kg (intraperitoneal), >80,000 mg/kg (intravenous), >800,000 mg/kg (oral) and >400,000 mg/kg (subcutaneous) and classified as class 4 chemicals whereas adverse effects of this compound are hepatotoxicity, arrhythmia and cardiac failure which can be altered with calibrated doses. Pharmacokinetic analysis proved that thymohydroquinone is BBB and CNS permeable; its low toxicity and mutagenic properties indicate that is a promising candidate in the treatment of these disorders and might be useful in the rejuvenation of BBB.

## Conclusion

All molecular drug targets including human IFN-γ, IL-1β, TNF-α, resisitin, VCAM-1 and MMP-9 demonstrated inhibitory interaction with thymohydroquinone by forming stable hydrogen bonds that prevented the targets from exerting their normal activity. Furthermore, this compound has high acute toxicity as analyzed through different administration routes with lower life-threatening side effects, which can be mitigated through nanocarriers. Thymohydroquinone has an efficient pharmacokinetic profile and could be the next promising single-drug candidate for the treatment and rejuvenation of these neurological anomalies. To our current knowledge, thymohydroquinone has multifaceted activity against these disorders which were assessed for the first time in this study and achieved efficacious results.

## Future perspective

There is a strong need to develop rodent or human BBB models to study the effect of these pathogenic proteins and further corroborate the findings of the current study. This will allow the scientific community to investigate the effectiveness of thymohydroquinone in these models and determine whether could be used as a vehicle to understand drug pharmacokinetics and their therapeutic influence on BBB rehabilitation before subjecting it to clinical trials.

**Summary Points**

- The onset of neuropsychiatric and neurodegenerative disorders (NNDs) is related with the deterioration of blood–brain barrier (BBB) which is facilitated by five pathogenic proteins markers; human IFN-γ, IL-1β, TNF-α, resisitin, VCAM-1 and MMP-9, respectively.
- Thymohydroquinone is assumed to be active against different NNDs but has not been evaluated in BBB-associated NNDs.
- An elaborate drug discovery research was conducted, involving observations of molecular interaction and stability of thymohydroquinone with different BBB-deteriorating protein markers, acute toxicity, adverse effects and pharmacokinetics of this ligand were predicted.
- Our study revealed that thymohydroquinone is a potent inhibitor of the BBB-disrupting proteins and show adequate interaction with all molecular targets responsible for BBB-associated NNDs.
- This compound possesses appreciable binding and ligand efficiency with a promising inhibitory constant value.
- It possesses BBB and central nervous system permeability with a high acute toxicity profile.
- The rare adverse effects associated with thymohydroquinone are hepatotoxicity, arrhythmia and cardiac failure.
