# Supplementary material for: In silico study of thymohydroquinone interaction with blood–brain barrier disrupting proteins
Source: Future Sci OA. 2020 Sep 25;6(10):FSO632. doi: 10.2144/fsoa-2020-0115 (PMC7720366; doi:10.2144/fsoa-2020-0115)
Supplement: Supplementary file 1 [file fsoa-06-632-s1.docx]

| **Docking Molecule** | **Spacing** | **Size** | | | **Center** | | |
| --- | --- | --- | --- | --- | --- | --- | --- |
|  |  | **X** | **Y** | **Z** | **X** | **Y** | **Z** |
| Human Interferon-Gamma (1HIG) | 0.786 | 40 | 40 | 40 | 19.521 | 27.476 | 26.738 |
| Resisitin (1RFX) | 0.803 | 40 | 40 | 40 | -16.934 | 55.87 | 15.387 |
| Interleukin 1 beta F101W (1T4Q) | 0.614 | 40 | 40 | 40 | -22.111 | 14.081 | -1.640 |
| Tumor Necrosis Factor-Alpha (1TNF) | 0.575 | 40 | 40 | 40 | 34.058 | 58.769 | 42.803 |
| Vascular cell adhesion molecule 1 (VCAM-1) (1VSC) | 0.536 | 40 | 40 | 40 | 29.529 | 14.694 | 27.329 |
| Matrix Metalloproteinase-9 (6ESM) | 0.531 | 40 | 40 | 40 | -0.438 | 43.009 | 14.809 |

**Supplementary Table 1.** Docking Parameters and Grid Box Configuration for Autodock 4

**Supplementary Table 2.** Other Predicted Active Site Subunits of Protein Targets

| **No** | **Receptor** | **Predicted Active Site Subunits** |
| --- | --- | --- |
| 1 | Human Interferon-Gamma (1HIG) | **Binding Site 2:** TYR98, SER99, VAL100, LEU9, VAL105, THR96, ALA109, GLU112, LYS108, VAL116, LEU113, LEU120, GLU119, GLN106, ILE110, ARG107, THR101, ASN104, ASN97, LYS94, GLN115, ASP91, ASP90, PHE92, GLU93  **Binding Site 3**: LYS88, PHE92, ASP91, LEU95, PHE81, PHE82, LYS43, GLN46, ASN83, SER47, ILE44, LYS80, VAL50, ILE49, TYR53, MET77, LEU33 |
| 2 | Resisitin (1RFX) | **Binding Site 2:** THR51, ALA52, VAL53, GLU69, GLU70, LYS71, CYS47, PRO48, GLU49, GLY50, SER46, LEU54, ILE67  **Binding Site 3**: ALA27, ILE28, ILE31 |
| 3 | Interleukin 1 beta F101W (1T4Q) | **Binding Site 2:** VAL85, ASN89, LYS94, MET95, GLU96, PHE99, VAL47, ALA59, VAL100, LYS92, TYR90, TYR68, ARG98, LYS93, ASP86, LYS88  **Binding Site 3**: TYR24, LEU82, SER84, GLU83, ARG98, GLN81, LEU80, GLU25, THR79, PHE133, LEU134, PRO78, LEU69, VAL132, LEU26, LEU18, ILE122, GLY22, SER21, PRO23, PRO131, LYS74, VAL72 |
| 4 | Tumor Necrosis Factor-Alpha (1TNF) | **Binding Site 2:** LYS65, TYR141, LEU142, LEU143  **Binding Site 3**: PRO20, ALA22, GLU23, GLY24, PHE144 |
| 5 | Vascular cell adhesion molecule 1 (VCAM-1) (1VSC) | **Binding Site 2:** GLU66, GLN85, GLU87, ARG123, GLN38, HIS67, SER68, VAL86, TYR119, PRO120, ARG146, LYS147, ASP122, ILE88, LEU12, ILE177, TYR89  **Binding Site 3:** GLU4, THR5, LYS82 |
| 6 | Matrix Metalloproteinase-9 (6ESM) | **Binding Site 2:** LEU222, HIS226, ALA242, LEU243, TYR245, MET247, ARG249, TYR248, VAL223, PRO246, LEU188, GLN227, LEU187, ALA189, HIS190, GLY186, HIS230, TYR179, ALA191, HIS236, ALA135, VAL136, ASP139, ALA140, LEU132, LEU212, LEU220, ARG143, SER211, PHE221, SER219, GLY213, PHE250, LYS214, THR251, GLU252, GLY253, PRO255, LEU147, PRO254, ILE137, TRP210  **Binding Site 3:** GLN126, ARG162, VAL167, GLN169, ASP177, PHE192, ILE198, ASP201, HIS175, GLY176, HIS203, ASP165, GLY197, GLN199, TYR179, HIS190, ALA191, ALA164, GLY195, PRO196, TRP124, GLY178 |

**Supplementary Table 3.** Chain Selection and Protein Sequence of Protein Targets

| **No** | **Receptor** | **Chain Selected for Docking** | **Protein Sequence** |
| --- | --- | --- | --- |
| 1 | Human Interferon-Gamma (1HIG) | Chain A | QDPYVKEAENLKKYFNAGHSDVADNGTLFLGILKNWKEESDRKIMQSQIVSFYFKLFKNFKDDQSIQKSVETIKEDMNVKFFNSNKKKRDDFEKLTNYSVTDLNVQRKAIHELIQVMAELSPAAKTGKRKRSQMLFRG |
| 2 | Resisitin (1RFX) |  | SSMPLCPIDEAIDKKIKQDFNSLFPNAIKNIGLNCWTVSSRGKLASCPEGTAVLSCSCGSACGSWDIREEKVCHCQCARIDWTAARCCKLQVA |
| 3 | Interleukin 1 beta F101W (1T4Q) |  | APVRSLNCTLRDSQQKSLVMSGPYELKALHLQGQDMEQQVVFSMSFVQGEESNDKIPVALGLKEKNLYLSCVLKDDKPTLQLESVDPKNYPKKKMEKRFVWNKIEINNKLEFESAQFPNWYISTSQAENMPVFLGGTKGGQDITDFTMQFVSS |
| 4 | Tumor Necrosis Factor-Alpha (1TNF) |  | VRSSSRTPSDKPVAHVVANPQAEGQLQWLNRRANALLANGVELRDNQLVVPSEGLYLIYSQVLFKGQGCPSTHVLLTHTISRIAVSYQTKVNLLSAIKSPCQRETPEGAEAKPWYEPIYLGGVFQLEKGDRLSAEINRPDYLLFAESGQVYFGIIAL |
| 5 | Vascular cell adhesion molecule 1 (VCAM-1) (1VSC) |  | FKIETTPESRYLAQIGDSVSLTCSTTGCESPFFSWRTQIDSPLNGKVTNEGTTSTLTMNPVSFGNEHSYLCTATCESRKLEKGIQVEIYSFPKDPEIHLSGPLEAGKPITVKCSVADVYPFDRLEIDLLKGDHLMKSQEFLEDADRKSLETKSLEVTFTPVIEDIGKVLVCRAKLHIDEMDSVPTVRQAVKELQVD |
| 6 | Matrix Metalloproteinase-9 (6ESM) |  | FEGDLKWHHHNITYWIQNYSEDLPRAVIDDAFARAFALWSAVTPLTFTRVYSRDADIVIQFGVAEHGDGYPFDGKDGLLAHAFPPGPGIQGDAHFDDDELWSLGKGVGYSLFLVAAHQFGHALGLDHSSVPEALMYPMYRFTEGPPLHKDDVNGIRHLYG |

**Supplementary Table 4.** Side Effects of Thymohydroquinone

Pa: Pharmacological Active Pi: Pharmacological Inactive

| **Pa** | **Pi** | **Side Effect** |
| --- | --- | --- |
| 0.761 | 0.075 | Hepatotoxicity |
| 0.419 | 0.171 | Arrhythmia |
| 0.313 | 0.159 | Cardiac failure |
